# Multidrug Resistant *Pseudomonas aeruginosa* in Clinical Settings: A Review of Resistance Mechanisms and Treatment Strategies

**DOI:** 10.3390/pathogens13110975

**Published:** 2024-11-07

**Authors:** Beth Schwartz, Katherine Klamer, Justin Zimmerman, Pramodini B. Kale-Pradhan, Ashish Bhargava

**Affiliations:** 1 Department of Internal Medicine, Henry Ford St. John Hospital, Detroit, MI 48236, USA; 2 Thomas Mackey Center for Infectious Disease, Henry Ford St. John Hospital, Detroit, MI 48201, USA; kklamer1@hfhs.org; 3 Department of Pharmacy Practice, Eugene Applebaum College of Pharmacy and Health Science, Wayne State University, Henry Ford St. John Hospital, Detroit, MI 48201, USA; justinzimmerman1027@wayne.edu (J.Z.); pkale@wayne.edu (P.B.K.-P.); 4 School of Medicine, Wayne State University, Detroit, MI 48021, USA

**Keywords:** *Pseudomonas aeruginosa*, drug resistant, multidrug resistance, infectious diseases

## Abstract

*Pseudomonas aeruginosa* is causing increasing concern among clinicians due to its high mortality and resistance rates. This bacterium is responsible for various infections, especially in hospital settings, affecting some of the most vulnerable patients. *Pseudomonas aeruginosa* has developed resistance through multiple mechanisms, making treatment challenging. Diagnostic techniques are evolving, with rapid testing systems providing results within 4–6 h. New antimicrobial agents are continuously being developed, offering potential solutions to these complex clinical decisions. This article provides a review of the epidemiology, at-risk populations, resistance mechanisms, and diagnostic and treatment options for *Pseudomonas aeruginosa*.

## 1. Introduction

Resistant *Pseudomonas aeruginosa* (*P. aeruginosa*) infections are a significant public health issue, with high mortality and morbidity rates, increased resource utilization, and costs [1]. *P. aeruginosa*, is an opportunistic Gram-negative, rod-shaped, non-spore-forming, motile bacterium commonly present in soil, plants, and diverse water sources, including rivers, sewage treatment facilities, untreated hospital effluents, swimming pools, and hot tubs. Mucoid *P. aeruginosa* can survive even in treated swimming pools [2]. The untreated hospital wastewater is a significant source of multidrug-resistant (MDR) *P. aeruginosa* for the environment [3]. The organism’s adaptability to its environment and various antibiotics has made it a notable concern in clinical settings.

*P. aeruginosa* is estimated to have a prevalence of 7.1–7.3% among all healthcare-associated infections [4,5]. According to the International Network for Optimal Resistance Monitoring (INFORM) database, current rates of MDR *P. aeruginosa* range from 11.5 to 24.7% of total *P. aeruginosa* [6]. Hospital-acquired infections from six bacterial strains, including MDR *P. aeruginosa*, were reported to have increased by 20% during the COVID-19 pandemic, according to the Centers for Disease Control and Prevention (CDC) [7]. The CDC lists MDR *P. aeruginosa* as a “serious threat” [8]. In 2017, *P. aeruginosa* alone was responsible for an estimated $767 million in healthcare spending in the United States (US) [8]. Hospital-acquired MDR *P. aeruginosa* infections resulted in a national cost exceeding $492 million [9]. Compared to non-MDR *P. aeruginosa* infection, MDR *P. aeruginosa* infection is associated with a 20% higher mortality rate, an excess cost of over $20,000, and increased length of stay and readmission rates [1].

## 2. Epidemiology in at-Risk Populations 

**Figure 1:**
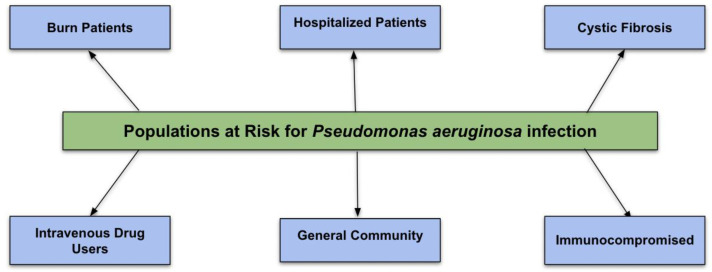
Flowchart of populations at risk for *P. aeruginosa* infection.

The at-risk population for *P. aeruginosa* infections includes patients with structural lung diseases such as cystic fibrosis, intravenous drug users, immunocompromised hosts, and burns patients (Figure 1). Community-acquired *Pseudomonas* infections are rare.

### 2.1. Hospitalized Patients

The prevalence of *P. aeruginosa* in hospital-acquired infections is estimated to be between 7.1% and 7.3% [4,5] According to a report from 2015 to 2017 by the National Healthcare Safety Network, *P. aeruginosa* is the fourth most common cause of all healthcare-associated infections [10]. In the intensive care unit (ICU), *P. aeruginosa* infection is estimated to account for 16.2% of all infections and 23% of all ICU-acquired infections. It is responsible for 9.62% of all hospital-acquired pneumonia (HAP) cases [11]. Additionally, *P. aeruginosa* is the second most common cause of ventilator-associated pneumonia (VAP), contributing to 10–20% of VAP cases [10]. Mortality secondary to *P. aeruginosa* VAP appears to be even higher, with estimates ranging from 32 to 42.8% [12,13,14].

*P. aeruginosa* bloodstream infections (BSIs) have a mortality rate of 43.2% to 58.8% [14,15,16]. They have a higher mortality rate compared to *Staphylococcus aureus* and other Gram-negative bacteria. MDR strains complicate 16.7% to 28% of these infections and are linked to higher mortality rates [14,16]. Risk factors for increased mortality in MDR *P. aeruginosa* BSI include respiratory origin, neutropenia, and insufficient empiric treatment [17].

*P. aeruginosa* causes around 10% of catheter-associated urinary tract infections (CAUTIs) and 16% of urinary tract infections in ICU patients [18,19]. In South Korea, a study of 260 patients with MDR *P. aeruginosa* bacteriuria found a 16.9% all-cause mortality rate within 12 months, with 31.8% of the deaths attributed to the infection itself [20].

*P. aeruginosa* accounts for approximately 5.7% of surgical site infections (SSIs) [4]. A study of 3494 patients at an Italian academic hospital revealed a 4.43% SSI rate, with a 1.34% multi-drug-resistant organism (MDRO) rate. *P. aeruginosa* caused 7.89% of the MDRO SSIs [21].

### 2.2. Cystic Fibrosis 

Cystic fibrosis impacts electrolyte transport and cell signaling, leading to the production of thickened mucus. The thick mucus hinders the respiratory cilia’s ability to clear pathogens, resulting in bacterial overgrowth [22]. The Cystic Fibrosis Foundation Annual 2021 Data Report shows a decrease in *P. aeruginosa* infections among patients, with a reduction in MDR *P. aeruginosa* infections from 4.2% in 2020 to 3.5% in 2021, especially in older adolescent and young adult patients [23].

### 2.3. Burn Patients 

*P. aeruginosa* is the most common Gram-negative bacteria that causes infections in hospitalized burn victims [24,25,26,27]. Its prevalence in burn units ranges from 13% to 50% in different international hospitals [28,29,30]. In pediatric burn units, approximately 86% of sepsis deaths are due to MDR *P. aeruginosa* [25]. 

### 2.4. Intravenous Drug Use 

Patients who abuse intravenous drugs are at the highest risk for *P. aeruginosa* infective endocarditis (IE). A study of 299 patients found that drug abusers had a significantly higher incidence of *P. aeruginosa* IE compared to non-drug abusers [31]. Most cases are due to using contaminated water or equipment during drug abuse [32]. Intravenous drug use is an independent risk factor for MDR *P. aeruginosa*. In a study of 358 patients with *P. aeruginosa* bacteremia, drug abusers were over 13 times more likely to have MDR *P. aeruginosa* bacteremia compared to non-abusers [33]. 

### 2.5. Immunocompromised States 

#### 2.5.1. Febrile Neutropenia

*P. aeruginosa* is the third most common cause of Gram-negative bacteremia in febrile neutropenic patients. A study on over 9,000 patients who underwent stem cell transplantation for acute leukemia analyzed outcomes following the first instance of BSI after a period of neutropenia. Out of the 1,424 patients who developed BSI, those with *P. aeruginosa* BSIs had the highest mortality rates at seven and thirty days, with a 7-day mortality of 16.7% and a 30-day mortality of 26.7% [34]. A study investigated mortality related to MDR *P. aeruginosa* in 190 neutropenic patients with *Pseudomonas* BSI. A mortality rate of 42.6% was reported, with a BSI-related mortality rate of 37.1% [35]. Other studies have reported a mortality rate as high as 70% for neutropenic patients with MDR *P. aeruginosa* bacteremia [36].

#### 2.5.2. Transplant Recipients

Transplant recipients are at high risk for *Pseudomonas* infections, especially in the three months following the transplant [37]. Mortality estimates for transplant recipients with *P. aeruginosa* BSIs can be as high as 50% [37]. In transplant recipients, 43% of isolates were multidrug-resistant (MDR), compared to 18% in non-transplant recipients [37]. MDR *P. aeruginosa* is often associated with nosocomial pneumonia in kidney and liver transplant recipients, with reported rates of 50–65% in this group [38,39].

#### 2.5.3. General Community Infections

Community-acquired pneumonia caused by *P. aeruginosa* is rare, with a prevalence of approximately 4.2% [40]. This bacterium can also cause otitis externa (swimmer’s ear) and hot tub folliculitis. Additionally, it has been reported to cause keratitis, especially with contaminated contact lens solution or tap water.

Community infections with MDR *P. aeruginosa* are rare. A study of 60 patients with community-acquired *P. aeruginosa* BSIs found that most were sensitive to key antibiotics: 100% to meropenem, 95% to ceftazidime, and 95% to piperacillin/tazobactam [41].

## 3. Risk Factors for Infection

The risk factors for *P. aeruginosa* infection are extensive and indicate an existing frailty, severe illness, or immunocompromised state. Scoring systems have been investigated to assess the risk of developing a carbapenem-resistant (CR) *P. aeruginosa* infection. A cohort study identified CR *P. aeruginosa* infection in the past 30 days, hospitalization in the past six months, presence of a tracheostomy, nursing facility residence, and carbapenem, cephalosporin, or fluoroquinolone use in the past 30 days. A separate scoring system was also examined to assess extensive β-lactam resistance which included resistance to carbapenems, ceftazidime, and piperacillin-tazobactam [42]. The study identified similar risk factors, with some exceptions. It was found that carrying a central venous catheter and using carbapenem antibiotics in the previous 30 days were also linked to β-lactam resistance. A different study examined COPD patients hospitalized for CAP. This system assigned points to prior *P. aeruginosa* infection or colonization, hospitalization in the last 12 months, and bronchiectasis. The authors suggested that using this scoring system, PAS-COPD could reduce antibiotic overuse from 54% to 6% [43]. A meta-analysis of 54 studies indicated that previous ICU or hospital admission and antibiotic use were the strongest risk factors for resistance [44]. 

## 4. Pathogenesis

### 4.1. Twitching Motility

Type IV pili and the flagella allow the bacteria to traverse across the host cell’s surface via a “twitching” motion that is unique to this pili type. The pili retract and extend, creating the movement via an undescribed mechanism. This movement also allows *P. aeruginosa* to develop a mature biofilm and allow the bacteria to further adhere to the host cell [41,45]. With the abnormal movement of the pili, the bacteria create a dense, mushroom-shaped biofilm that does not adhere as effectively to surfaces as the wild-type strain of *P. aeruginosa* [42,46]. 

### 4.2. Type III Secretion System

The type III secretion system (T3SS) is a needle-like complex that injects exoenzyme effectors into the eukaryotic host cells’ cytosol. The well-characterized effectors are ExoU, ExoS, ExoT, and ExoY. The pathogenic effects vary with effector type when interacting with a eukaryotic co-factor. Effects include disrupting the host’s cytoskeleton, leading to apoptosis, disrupting cGMP signaling, and effectively causing cell lysis and inhibiting inflammasome activation [43]. ExoU leads to cell lysis and is commonly associated with pneumonia infections [44,47]. ExoU leads to cell lysis and is commonly associated with pneumonia infections [48]. All *P. aeruginosa* have genes for T3SS; however, not all strains can produce the proteins that compose the system. Those strains that can produce T3SS may cause more severe clinical consequences in humans [28,32]. A 248-patient study conducted at Yale New Haven Hospital found that a T3SS-positive phenotype was associated with a higher risk of infections in patients with culture-positive *P. aeruginosa* [49,50]. 

### 4.3. Pyocyanin

This pigment, which gives *Pseudomonas* the classic blue-green color, also serves a role in the organism’s virulence. The type II secretion system secretes this pigment and causes oxidative stress via the disruption to the electron transport system of the host cell. It can also cause disruptions in the immune system by inhibiting the release of IL-2 [45,51]. 

## 5. *Pseudomonas* Resistance Mechanisms

**Figure 2:**
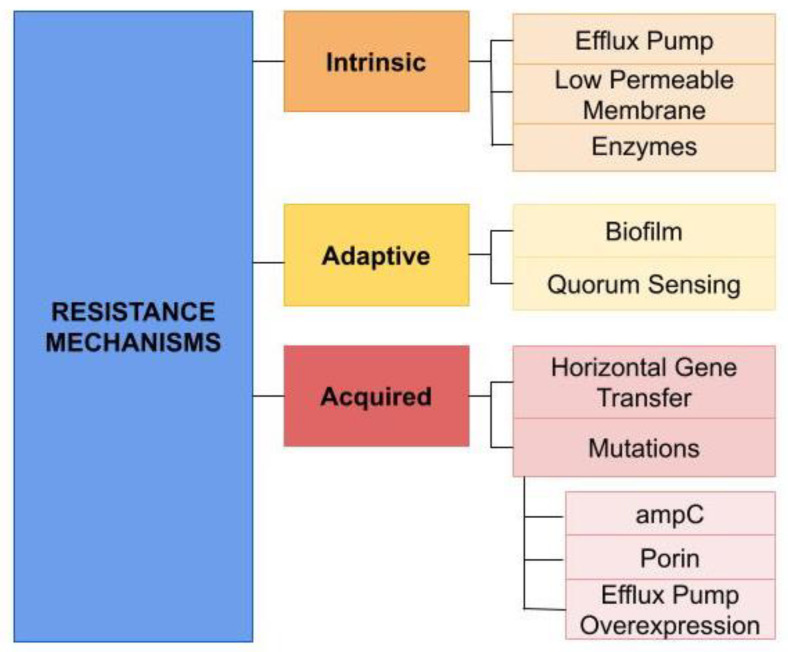
Flowchart of important mechanisms of *P. aeruginosa*.

*P. aeruginosa* displays various resistance mechanisms, commonly classified into intrinsic, acquired, and adaptive (Figure 2). Intrinsic resistance includes efflux pumps, low permeability of the outer membrane, and enzymes to deactivate antibiotics. Acquired resistance includes horizontal gene transfer or mutations confer resistance [49]. Lastly, adaptive resistance includes biofilm production in the lungs of infected patients, which shields bacteria from antibiotics [49].

### 5.1. Intrinsic Resistance

#### 5.1.1. Efflux Pump Systems

These serve as a defense mechanism by expelling harmful substances, including antibiotics from bacterial cells [12].

#### 5.1.2. Low Permeable Membrane

For Gram-negative organisms the hydrophobic lipid bilayer in combination with pore-forming proteins allows for the selection of certain-sized molecules into the cell interior [52].

#### 5.1.3. Antibiotic Degrading Enzymes

*P. aeruginosa* has multiple enzymes that are common in most isolates of *P. aeruginosa*. *AmpC* Beta-Lactamases are chromosomally encoded enzymes that give resistance to penicillins, second- and third-generation cephalosporins, and cephamycins [53]. Aminoglycoside resistance can come from a one or a combination of the enzymes aminoglycoside phosphoryltransferase (APH), aminglycoside acetyltransferance (AAC) or aminoglycoside nucleotidyltransferase (ANT) [54]. Chloramphenicol resistance is mainly from chloramphenical acetyltransferases or, in some cases, by chloramphenicol phosphotransferase [55].

### 5.2. Adaptive Resistance

#### 5.2.1. Biofilms

Alginate is an exopolysaccharide that forms the biofilm of *P. aeruginosa* and that contributes to the microbe’s ability to adhere to surfaces. This creates a stronger surface attachment and increased antibiotic resistance due to biofilms. As biofilm depth increases, cellular metabolic activity decreases, providing further protection to bacteria against antibiotics and increasing the likelihood of recurrent infection [56]. 

#### 5.2.2. Quorum Sensing

Through this process, chemical signals called autoinducers increase in concentration when cell density is increased. When enough autoinducers accumulate, the bacteria respond by changing their gene expression and coordinating their behavior. Through this cascade activation, quorum sensing enhances biofilm formation [32].

#### 5.2.3. Carbapenem-Resistance (CR)

CR is mediated mainly by mutations within chromosomes that change the bacterial porins, efflux pump activity, and allow for expression of β-lactamase activity [57]. However, carbapenemases, encoded on plasmids, can also mediate resistance [57,58]. Carbapenemases are uncommon in *P. aeruginosa* isolates in the United States; this can be attributed to the fact that carbapenemase testing for DTR *P. aeruginosa* is not as critical as carbapenemase testing for CRE clinical isolates in United States hospitals [59]. However, the Infectious Diseases Society of America (IDSA) encourages clinical microbiology laboratories to perform AST for MDR and DTR *P. aeruginosa* isolates against newer β-lactam agents (i.e., ceftolozane- tazobactam, ceftazidime–avibactam, imipenem–cilastatin–relebactam, and cefiderocol) [60].

### 5.3. Acquired Resistance

Acquired resistances through horizontal gene transfers and mutations have been playing a pivotal role in the resistance of *P. aeruginosa.* The culmination of the acquiring mechanisms has caused common resistant *Pseudomonas* types such as extended-spectrum B-lactamases and carbapenem resistance.

#### 5.3.1. AmpC Mutation

The mutational mechanism that mainly drives β-lactam resistance and involves overproduction of a chromosomal cephalosporinase known as *AmpC*. Inactivation of *dacB* is the leading cause of mutational *AmpC* activation [61]. 

##### Horizontal Gene Transfer

Another mechanism of resistance is through horizontal gene transfer. *P. aeruginosa* can acquire a wide variety of metallo-β-lactamase genes such as *IMP, VIM, SPM, GIM, NDM* and *FIM*. These encode enzymes capable of hydrolyzing a broad range of β-lactam antibiotics [62]. 

##### Extended-Spectrum B-Lactamases (ESBL)

Acquired resistance genes are primarily advantageous for bacterial survival, providing antibiotic resistance, predominantly against B-lactams and aminoglycosides [32]. ESBLs are transmitted through plasmids and result in resistance to penicillin, cephalosporins, aztreonam, and, at times, carbapenems. Various ESBL families have been identified in *P. aeruginosa* strains, including *Pseudomonas* extended resistance (*PER*) enzymes, Vietnamese extended-spectrum beta-lactamase (VEB), Guiana extended-spectrum beta-lactamase (*GES*), *TEM, SHV*, and *CTX-M* [32]. Oxacillinase β-lactamases may have weak sensitivity to clavulanic acid. Carbapenemase-hydrolyzing oxacillinases can be acquired or spontaneously occur. Metallo-carbapenemase enzymes include Verona integron-encoded metallo-B-lactamase (*VIM*), Imipenemase (*IMP*), and New Delhi metallo-B-lactamase (*NDM*) families [32]. *NDM* enzymes can also confer aminoglycoside and fluoroquinolone resistance genes [32]. *Klebsiella pneumoniae* carbapenemase (*KPC*) has also been found in some *P. aeruginosa* isolates [32]. *Pseudomonas* produces *AmpC* β-lactamases, providing resistance to penicillin, certain cephalosporins (excluding cefepime), and other β-lactam antibiotics. Elevated levels of *AmpC* may hinder the detection of ESBLs through phenotypic testing [63].

#### 5.3.2. Porin Mutation

Porin proteins such as *OprD* create channels within the *P. aeruginosa* membrane for hydrophilic antibiotics to enter. Mutations in the porins confer resistance to carbapenem antibiotics by reducing bacterial membrane permeability. This mutation confers high- level resistance to carbapenems, particularly imipenem [64].

#### 5.3.3. Efflux Pump Overexpression

Mutations in regulatory genes such as *mexR, nalB, nalC* or *nalD* disrupt normal efflux pump expression and cause overexpression, allowing the cell to expel antibiotics at a faster rate than normal. The expression of *mexXY* efflux proteins increases *P. aeruginosa* resistance to antibiotics such as novobiocin, chloramphenicol, β-lactams, lincomycin, macrolides, and fluoroquinolones in addition to the antibiotics that the wild type is inherently resistant to. Combinations of proteins encode different resistances; for example, *mexAB-OprM* is known for its broad spectrum of resistance to cephalosporins, ticarcillin, and β-lactams [12].

## 6. Diagnostics

Current culture interpretations of *P. aeruginosa* are Gram-negative rods straight to slightly curved in a Gram-stain. They appear on blood agar with a pearlescent sheen with irregular margins but can also appear mucoid. With pyocyanin production, they also produce a blue-green pigment, making them appear dark-colored on blood agar and blue- green on Mueller Hinton. They do not ferment lactose, making them clear-colored on MacConkey agar, and are positive for oxidase. The aroma is unique, often described as having a “grape-like” or “fresh tortilla” scent. Confirmatory testing involves incubating the organism at 42°C for 24–48 h. An antibiotic panel can take 48–72 h to complete, requiring providers to administer broad-spectrum coverage until then [65].

Selective and differential agars have been developed that can be used for rapid identification and selection of potential Gram-negative ESBLs, CREs, and other antibiotics. This can provide preliminary results within 24 h of sample inoculation to the plate [66].

Lateral flow assays are another rapid method of detecting resistant organisms. They can detect antimicrobial resistance from a small sample volume. Multiple formats can detect numerous different resistances. They also require very little training and setup; results come within minutes [67].

Nucleic Acid Amplification (NAA) testing is one of the most common rapid identification methods. In cases of sepsis, samples can be taken directly from the bottle to perform polymerase chain reaction (PCR). Rapid identification of organisms and resistance genes can be achieved in approximately 4–6 h from the samples. PCR is the most common type of NAA for antimicrobial resistance, allowing the identification of many resistance mechanisms in patient samples.

Many microbiology laboratories use Matrix-Assisted Laser Desorption Ionization–Time-of-Flight (MALDI–TOF) to identify organisms. New algorithms for antibiotic resistance are being developed for MALDI–TOF. Biomarkers such as proteins, carbohydrates, lipids, and enzymatic activity can be used to detect specific resistance mechanisms in multiple species of bacteria [68].

## 7. Treatment for *Pseudomonas* Infections

### Pharmacologic Agents for Susceptible P. aeruginosa

Antibiotics with activity against *P. aeruginosa* include penicillins with a β-lactamase inhibitor (piperacillin–tazobactam, ticarcillin–clavulanate), broad spectrum cephalosporins with or without a β-lactamase inhibitor (cefepime, ceftazidime, ceftolozane–tazobactam, ceftazidime–avibactam), fluoroquinolones (levofloxacin, ciprofloxacin), carbapenems (imipenem, doripenem, meropenem), monobactams (aztreonam), and aminoglycosides (tobramycin, gentamicin, amikacin) [32].

## 8. Classes of Antibiotic Resistance

### 8.1. Multidrug Resistance

According to the IDSA 2024 Guidance on the Treatment of Antimicrobial-Resistant Gram-negative Infections “MDR *P. aeruginosa* is defined as *P. aeruginosa* not susceptible to at least one antibiotic in at least three antibiotic classes for which *P. aeruginosa* susceptibility is generally expected: penicillin’s, cephalosporins, fluoroquinolones, aminoglycosides, and carbapenems” [60].

### 8.2. Difficult to Treat

According to the IDSA 2024 Guidance on the Treatment of Antimicrobial-Resistant Gram-negative Infections “*Difficult to Treat* (DTR) is defined as *P. aeruginosa* exhibiting non-susceptibility to all of the following: piperacillin-tazobactam, ceftazidime, cefepime, aztreonam, meropenem, imipenem-cilastatin, ciprofloxacin, and levofloxacin” [60].

## 9. Newer Pharmacologic Agents Against Resistant *P. aeruginosa*

### 9.1. Ceftolozane-Tazobactam

Ceftolozane–tazobactam (C–T) is Food and Drug Administration (FDA) FDA approved for the treatment of complicated intra-abdominal infections (cIAIs), complicated urinary tract infections (cUTIs), HAP, and VAP. Ceftolozane is effective against a wide variety of Gram-negative and Gram-positive bacteria. Adding tazobactam enhances activity against Gram-negatives, including ESBL *Enterobacterales* and *P. aeruginosa*, as well as MDR *Pseudomonas*. It also improves activity against anaerobes, including *Bacteroides, Prevotella*, and *Clostridium* species. C–T has activity against *Streptococcus* species but has limited activity against *Staphylococcus* species [69]. 

Ceftolozane exhibits a high affinity for penicillin-binding protein (PBP) 3 and maintains a greater affinity for PBP1b than other β-lactams. Due to its reduced susceptibility to hydrolysis by *AmpC* β-lactamases produced by *P. aeruginosa* and as a weak substrate for *P. aeruginosa*’s efflux pump systems, ceftolozane is a promising agent to treat such infections. In addition to being resistant to efflux pumps, ceftolozane is also unaffected by loss of the *OprD* porin, a key resistance mechanism to carbapenems in *P. aeruginosa* [70]. 

ASPECT-cUTI is a phase 3, double-blind, double-dummy trial that compared C–T (1.5 g every 8 h IV) to levofloxacin (750mg every 12 h IV) in patients with cUTIs in 209 centers. The primary endpoint of composite cure in the microbiological modified intention to treat (MITT) group was non-inferior to levofloxacin [306/398 (76.9%) vs 275/402 (68.4%) (CI 2.3–14.6)] [66]. In both the per-protocol group and mMITT groups, C–T was superior to levofloxacin for microbiological eradication. This study led to its approval for use in cUTIs [71]. 

ASPECT-cIAI, a phase 3 double-blind trial in which patients with cIAI were randomized to receive either the combination of C–T (1.5 g every 8 h) and metronidazole (500 mg every 8 h) or meropenem (1 g every 8 h). The primary outcome was to demonstrate statistical noninferiority in the clinical cure rates at the test-of-cure (TOC) visit (24–32 days after therapy initiation) in the microbiological intent-to-treat (MITT), using a noninferiority margin of 10%. Baseline characteristics were similar; most infections were polymicrobial with MDR *Pseudomonas* in 52 patients. The study found the clinical cure rates were 83% vs. 87.3% (CI –8.9–0.54) in the MITT for C–T and meropenem, respectively, meeting the noninferiority criteria, which led to its approval for cIAI [72].

In another phase 3 double-blind controlled study (ASPECT-NP), high-dose C–T (3 g every eight hours) was compared to meropenem (1 g every eight hours) in patients with nosocomial pneumonia. The primary outcome was mortality at 28 days at a noninferiority margin of 10%. The key secondary endpoint was clinical cure at the TOC visit with a noninferiority margin of 12.5%. The overall mortality at 28 days was 24% vs. 25.3% (95% CI −5.1 to 7.4) in the ceftolozane-tazobactam and meropenem groups, respectively. The overall clinical cure was similar between C–T and meropenem groups (54.4% and 53.3% (95% CI −6.2–8.3), respectively). This study demonstrated that C–T was non-inferior to meropenem in patients leading to its approval for nosocomial pneumonia [73].

### 9.2. Ceftazidime-Avibactam

Ceftazidime is a third-generation cephalosporin with Gram-positive and Gram-negative activity, including anti-*Pseudomonal* activity [74]. The addition of β-lactam inhibitor avibactam decreases the minimum inhibitory concentration (MIC) in ESBLs, carbapenemases, metallo β-lactamases, and Ambler class C β-lactamases, thereby enhancing its activity, including anti-*Pseudomonal* activity. 

Despite the mechanism of action of ceftazidime–avibactam (CAZ–AVI) in targeting *P. aeruginosa*, resistance can still develop. Enzymatic deactivation can occur through mutations in class C (*AmpC*) and class D (*OXA*) β-lactamases. Specifically, mutations in the omega loop region of *AmpC* can widen the enzyme’s binding pocket, enhancing its ability to catalyze ceftazidime but reducing its affinity for avibactam. In vitro studies have shown that combining ceftazidime–avibactam with other antimicrobials can lead to synergistic effects (e.g., meropenem, amikacin, aztreonam, colistin, or fosfomycin). This suggests that combination therapy may effectively treat ceftazidime-resistant strains of *P. aeruginosa* [70].

The RECAPTURE program, which included two identical phase 3 trials (RECAPTURE 1 and 2), compared the effectiveness and safety of CAZ–AVI (2000 mg/500 mg every 8 h) and doripenem (500 mg every 8 h) in patients with cUTI, including acute pyelonephritis in a double-blind, double-dummy, parallel-group manner. The symptomatic resolution of symptoms by day five was noted in 276 of 393 (70.2%) vs 276 of 417 (66.2%) patients (95% CI, −2.39% to 10.42%) in the CAZ–AVI and doripenem groups, respectively. The combined symptomatic resolution and microbiological eradication at TOC was 71.2%, compared to 64.5% in the ceftazidime–avibactam and doripenem groups, respectively (95% CI, 0.30% to 13.12%). This met the non-inferiority margin of 10%. Microbiological eradication at TOC occurred in 77.4% and 71.0% (95% CI, 0.33% to 12.36%) of patients in CAZ–AVI and doripenem groups, respectively, consistent with the superiority of CAZ–AVI. 

In another phase 3 multicenter, open-label trial, patients with cUTI or cIAIs caused by *Enterobacterales* or *P. aeruginosa* resistant to ceftazidime were randomly assigned to receive CAZ–AVI (n = 165) or the best available therapy (BAT) (*n* = 168), with 97% of them receiving carbapenem [75]. Most infections were cUTIs, 93.5% versus 92.5% in the CAZ–AVI and comparator groups, respectively. The clinical cure rates were similar in both groups with CAZ–AVI (91%; 95% CI 85.6–94.7) and the BAT (91%; 95%CI 85.9–95.0). This study supports CAZ–AVI as an alternative to carbapenems in patients with ceftazidime-resistant *Enterobacterales* and *P. aeruginosa*.

In a phase 3 trial, data from two identical, randomized, double-blind phase 3 studies were combined to evaluate the efficacy and safety of CAZ–AVI plus metronidazole compared to meropenem in 1066 patients with cIAIs [76]. The primary endpoint was clinical cure at TOC visit 28–35 days after randomization, with a noninferiority margin of 12.5%. Clinical cure rates in the mMITT were 81.6% and 85.1% (95% CI −8.64 to 1.58), and clinically evaluable population were 91.7% and 92.5% (95%CI −4.61 to 2.89) in the ceftazidime–avibactam plus metronidazole and meropenem, respectively. The study’s results demonstrated the non-inferiority of CAZ–AVI to meropenem, leading to approval for treating cIAIs.

In a randomized, multinational, phase 3 double-blind, non-inferiority trial (REPROVE), CAZ–AVI (2000mg–500mg every 8 h) in patients with nosocomial pneumonia, including VAP, was compared to meropenem (1 g every 8 h) [77]. The primary endpoint was a clinical cure at the TOC visit (21–25 days after randomization) with a non-inferiority margin of 12.5%. In the mMITT population, common pathogens were *Klebsiella pneumoniae* (37%) and *P. aeruginosa* (30%), and 28% were not susceptible to ceftazidime. In the clinically modified intention-to-treat population, the cure rates were 68.8% and 73% (95% CI –10·8 to 2·5) in the CAZ–AVI and meropenem groups respectively. The clinically evaluable groups demonstrated similar cure rates in both groups. The results indicate that CAZ–AVI was equally effective as meropenem in treating nosocomial pneumonia.

Based on the trials above, ceftazidime–avibactam is an effective agent approved for cUTIs, cIAIs, and nosocomial pneumonia, including VAP.

### 9.3. Imipenem-Cilistatin-Relebactam

Imipenem–relebactam (IMI–REL) is effective against various Gram-negative organisms, especially those with resistance mechanisms. It enhances IMI’s effectiveness against IMI non-susceptible isolates, ESBL, KPC, and serine carbapenemase-producing Enterobacteriaceae and MDR *P. aeruginosa*. In vitro studies have shown a 2- to 128-fold reduction in MIC values for Enterobacteriaceae and an eightfold reduction for *P. aeruginosa* when REL is added to IMI. Conversely, REL has minimal impact on IMI activity against *Acinetobacter baumannii*, *Chryseobacteria*, and *Stenotrophomonas maltophilia*. The activity of IMI–REL is enhanced significantly against isolates producing Ambler class A ESBLs and KPC carbapenemases. However, the combination does not improve activity against isolates with class B metallo-β-lactamases like IMP, VIM, and NDM. Furthermore, IMI–REL has limited efficacy against class D β-lactamase-producing isolates, such as OXA-23-producing A. baumannii. The addition of REL provides minimal benefit for anaerobic bacteria, although some improvement in MIC values was noted against *Bilophila wadsworthia* and *Fusobacterium* species. However, *Bacteroides* species with decreased IMI susceptibility showed no response to adding REL. Limited data exists regarding its efficacy against Gram-positive bacteria [78]. 

The SMART surveillance program data indicated that 67% of *P. aeruginosa* isolates were susceptible to IMI alone, which increased to 90% with the addition of REL. The addition of REL led to a significant decrease in MIC90 values, with an eightfold shift in IMI non-susceptible isolates and a fourfold shift in IMI-susceptible isolates. REL, a potent inhibitor of *AmpC* β-lactamases, restored IMI susceptibility in nearly 90% of *P. aeruginosa* isolates, including 70% of IMI-resistant strains. The IMI–REL combination shows enhanced efficacy in vitro, especially against CR *P. aeruginosa*, due to the lack of an inoculum effect and efflux resistance [79].

A randomized, double-blind, phase 3 trial compared IV IMI–REL 500 mg/250 mg every 6 h (*n* = 31) plus a colistimethate sodium placebo or IV colistin (a loading dose to achieve 300 mg colistin base activity followed by maintenance doses up to 150 mg every 12 h) plus IMI 500 mg every 6 h (*n* = 16) in hospitalized patients with HAP, VAP, cIAI, or cUTI caused by IMI-non-susceptible but IMI–REL- and colistin-susceptible Gram-negative pathogens. The primary endpoint was the overall favorable response at the end of treatment in the modified microbiologic intent-to-treat (mMITT) population. The most common pathogens were *P. aeruginosa* (77%), 84% of patients had *AmpC*-producing pathogens, and 16% had KPC-producing strains The IMI–REL group had a 71% favorable overall response, compared to 70% in the colistin plus IMI group (difference: –7.3%; 90% CI: –27.5%, 21.4%). IMI–REL was effective against *P. aeruginosa*, achieving an 81% favorable response compared to 63% in the colistin plus IMIgroup. Clinical response rates on day 28 were 31% higher in the IMI–REL group, and all-cause mortality was 20% lower compared to the colistin plus IMI group.

Overall, IMI–REL provided a favorable safety profile, with lower rates of nephrotoxicity and fewer adverse events compared to colistin plus IMI, making it a strong option for the treatment of CR *P. aeruginosa* infections [80]. 

A second randomized, controlled, double-blind phase 3 trial compared IMI–REL with piperacillin-tazobactam in adults with HAP or VAP. The trial randomized 537 patients from 113 hospitals in 27 countries between January 2016 and April 2019. Patients received either IV IMI–REL 500 mg/250 mg (n = 266) or piperacillin–tazobactam 4 g/500 mg every 6 h (n = 269) for a duration of 7–14 days. The primary endpoint, day 28 all-cause mortality, showed noninferiority of IMI–REL compared to piperacillin–tazobactam, with a mortality rate of 15.9% in the IMI–REL group and 21.3% in the piperacillin–tazobactam group (adjusted treatment difference, −5.3%; 95% CI, −11.9% to 1.2%; *p* < 0.001). The study results showed that IMI–REL was non-inferior to piperacillin–tazobactam, leading to approval for treating HAP/VAP [81].

REL improves IMI’s effectiveness by inhibiting class A and C β-lactamases. However, it has limitations against some resistance mechanisms, such as metallo-β-lactamases and *strains with blaOXA-51*, highlighting the need for further research. Overall, IMI–REL is a significant advancement in treating antibiotic-resistant infections.

### 9.4. Meropenem-Vaborbactam

Meropenem–vaborbactam (MEV) is FDA-approved for cUTI (including pyelonephritis). Vaborbactam is a boronic acid-based serine β-lactamase inhibitor, which forms a covalent bond with the serine active site of β-lactamases. This mechanism provides enhanced stability against hydrolysis by serine β-lactamases. A study examining 467 isolates of *P. aeruginosa* found that 79% of the isolates were susceptible to meropenem alone. However, among meropenem non-susceptible isolates, the addition of vaborbactam showed limited improvement, with a 4-fold or more significant decrease in meropenem MICs observed in only a small proportion of isolates (6–9%). The lack of improvement in *P. aeruginosa* was attributed to non-β-lactamase-mediated mechanisms of resistance, including reduced *OprD* expression, increased efflux (e.g., *mexA, mexC, mexE*, and *mexX* overexpression), and alterations in porins [82].

A retrospective, multicenter US study examined patients receiving MEV for MDR gram-negative infections. Among 232 isolates, 78.6% were CRE. *Klebsiella pneumoniae* (53.5%) and *Escherichia coli* (25.3%) were the most common. The study tested two isolates of *P. aeruginosa* for susceptibility to MEV. The MIC50 for *P. aeruginosa* was 18 mg/L, with a range of 4/8 to 32/8 mg/L, indicating limited reduction in MIC values when vaborbactam was added to meropenem [83].

The pivotal clinical trials for MEV did not specifically include patients with infections caused by *P. aeruginosa*. TANGO I focused on cUTIs, including acute pyelonephritis, while TANGO II evaluated the efficacy of MEV in treating CRE infections. Both trials showed that the combination improved clinical cure rates, reduced mortality, and lowered nephrotoxicity compared to piperacillin–tazobactam and the BAT. However, these studies focused on Gram-negative pathogens like *Klebsiella pneumoniae* and *Escherichia coli* and did not evaluate their performance against *P. aeruginosa*. Therefore, clinicians should consider the limited data for *P. aeruginosa* infections when contemplating MEV as a treatment option [84,85].

### 9.5. Cefiderocol

Cefiderocol is a siderophore cephalosporin that binds to extracellular iron, which is essential for bacteria to proliferate, and then binds to PBPs, rendering its bactericidal effect [86]. In a phase 2 multinational, double-blind trial, patients with cUTI were randomly assigned to receive cefiderocol 2 g or IMI/cilastatin 1 g every 8 h in a 2:1 ratio [87]. The primary endpoint was a composite of clinical and microbiological responses at TOC. The secondary endpoints were microbiological, clinical, and safety responses. The composite response rates at TOC were reported as 72.6% and 54.6% (95% CI 8.2–28.9), respectively, meeting the pre-specified non-inferiority margins of 15%. The microbiological response rates at TOC were 73% and 56% (95% CI 6.92 to 27.58), respectively. The clinical response at TOC was 98% and 99% (95% CI −4.66 to 9.44). 

The CREDIBLE-CR study was a multinational, open-label, pathogen-focused phase 3 trial designed to evaluate the efficacy and safety of cefiderocol compared to the BAT for treating patients with CR Gram-negative infections [88]. A total of 152 adult patients aged 18 years and older, diagnosed with nosocomial pneumonia (HAP/VAP), BAIs, sepsis, or cUTIs, were included from 95 hospitals in 16 countries. Patients were randomly assigned to receive cefiderocol 2 g every 8 h (n = 101) or the BAT (n = 51). The study’s primary endpoint was achieving a clinical cure in patients, except for cUTIs, for which microbiological eradication was the primary endpoint. 

Overall, the clinical cure rate for cefiderocol was 66%, while the BAT group achieved a rate of 58%. Among patients with nosocomial pneumonia, 60% of those treated with cefiderocol achieved clinical cure compared to 63% in the BAT group. Clinical cure rates for patients with BSIs or sepsis were 70% for the cefiderocol group and 50% for the BAT. In cases of cUTI, cefiderocol demonstrated a higher clinical cure rate of 77% compared to 60% for the BAT. Microbiological eradication was achieved in 84% of cefiderocol-treated patients with cUTIs versus 69% in the BAT group. The most frequently identified pathogens included *A. baumannii* (n = 54), *K. pneumoniae* (n = 39), and *P. aeruginosa* (n = 22).

All-cause mortality on day 14 for patients with nosocomial pneumonia was 24% in the cefiderocol group and 14% in the BAT group. By day 28, the mortality rates had increased to 31% for cefiderocol, compared to 18% for the BAT. The cefiderocol group had higher mortality rates for patients with BSIs or sepsis: 17% at day 14 compared to 6% for BAT and 23% at day 28 versus 18%, respectively. In contrast, the mortality rates for patients with cUTIs treated with cefiderocol were 12% on day 14 and 15% on day 28, compared to 20% for the BAT at both time points. Among patients infected with *P. aeruginosa*, the mortality rate was 35% for cefiderocol, compared to 17% for the BAT. 

Cefiderocol has shown potential as an effective treatment for carbapenem-resistant severe Gram-negative infections, particularly in cUTIs, despite higher mortality rates in patients with nosocomial pneumonia and bloodstream infections [88].

In phase 3, a multi-national, double-blind non-inferiority trial, patients with nosocomial pneumonia were randomized to cefiderocol 2 g (n = 145) or meropenem 2 g (n = 147) every 8 h [89]. Linezolid 600 mg was administered to all patients every 12 h in an open-label manner. The primary endpoint was all-cause mortality at day 14 in the mITT, with secondary endpoints being clinical and microbiological outcomes at TOC. The all-cause mortality at day 14 was 12.4% in the cefiderocol group and 11.6% in the meropenem group, meeting the non-inferiority margin of 12.5% (95% CI −6.6 to 8.2, *p* = 0.002). On day 28, the all-cause mortality rate was similar in both groups. The clinical cure rates reported were 65% for the cefiderocol group and 67% for the meropenem group, with similar microbiological eradication rates between the two groups.

Based on the above studies, Cefiderocol is FDA-approved for cUTIs and nosocomial pneumonia. While cefiderocol demonstrated similar efficacy to traditional therapies (e.g., extended-infusion meropenem, imipenem–cilastatin), there are no direct comparative studies with newer agents like C–T, CAZ–AVI, or IMI–REL. Cefiderocol may be considered when other β-lactams are unavailable or ineffective. However, current expert opinion favors newer β-lactam/β-lactamase inhibitors over cefiderocol as first-line therapy for MDR/DTR *P. aeruginosa* [90].

### 9.6. Colistin 

Colistin is a member of the polymyxin family, specifically polymyxin E. It serves as a last-line option in the treatment of MDR *P. aeruginosa* due to concerns about nephrotoxicity and neurotoxicity. Even with these concerns, colistin can serve as a last-resort therapy as resistance to first-line anti-pseudomonal antibiotics rises. Colistin disrupts the bacterial cell membrane, binding to the anionic lipopolysaccharides in Gram-negative bacteria’s outer membrane. Colistin has a narrow spectrum, mainly targeting Gram-negative bacteria. It is effective against organisms such as *Acinetobacter* spp., *Klebsiella* spp., *Enterobacter* spp., and nearly all strains of *P. aeruginosa* [91].

Colistin is highly effective but has significant toxicity concerns, primarily nephrotoxicity and neurotoxicity. Nephrotoxicity is present in up to 50% of cases but tends to be mild to moderate and reversible. Neurotoxicity is rare but may cause muscle weakness, vertigo, confusion, ataxia, and, in severe cases, respiratory failure.

Acquired resistance to colistin in *P. aeruginosa* primarily arises from modifications to lipid A, the lipid component of lipopolysaccharides in the bacterial outer membrane. This resistance typically results from the addition of one or two 4-amino-l-arabinose (l-Ara4N) molecules to the phosphate groups of lipid A [92]. There is a lack of randomized controlled trials assessing the efficacy of colistin in treating *P. aeruginosa* bloodstream infections. Therefore, colistin is used to treat confirmed cases of MDR *P. aeruginosa*. Empirical treatment is discouraged due to the risk of selecting resistant strains [91].

In a clinical trial, the effectiveness of C–T against MDR *P. aeruginosa* infections was compared with a colistin-based regimen [93]. The study found that patients treated with C–T had a significantly higher clinical cure rate than those receiving colistin-based regimens (77% vs. 57%; *p* = 0.005; 95% CI, 1.32–4.79). There were no significant differences in outcomes such as mortality, readmission rates, microbiological eradication, or length of stay. Acute kidney injury was significantly less common in patients receiving C–T than colistin (15% vs. 41%; *p* < 0.001; 95% CI, 0.12–0.51).

Colistin can be used to treat MDR *P. aeruginosa* after thoroughly evaluating the risks, benefits, and availability of other potential treatments. Colistin is considered a last-resort option for multidrug-resistant *P. aeruginosa*. Further studies are necessary to understand its role in therapy.

## 10. Clinical Recommendations

MDR *P. aeruginosa* infections present a challenge to clinicians. New combinations of β-lactam/β-lactamase inhibitors and carbapenem/carbapenemase inhibitors have been developed over the past several years. The IDSA has issued guidelines for treating Gram-negative infections [60].

*P. aeruginosa* strains are often found in cUTIs, cIAIs, and nosocomial pneumonias (e.g., HAP/VAP). The IDSA guidance recommends using traditional β-lactams such as piperacillin-tazobactam, ceftazidime, cefepime, aztreonam, and carbapenems for susceptible *P. aeruginosa*. If *P. aeruginosa* isolates are resistant to carbapenems but susceptible to traditional β-lactams, it is recommended to use traditional β-lactams. However, if the *P. aeruginosa* isolates are resistant to both carbapenems and traditional β-lactams, consider using the newer β-lactam/β-lactamase inhibitors and carbapenem/carbapenemase inhibitors such as C-T, CAZ-AVI, IMI-REL, depending on susceptibility. United States surveillance data showing culture and sensitivity results for *P. aeruginosa* with difficult-to-treat resistance shows non-carbapenem sensitive isolates as 90% sensitive to C-T, 85% sensitive to ceftazidime-avibactam, 86% sensitive to IMI-REL, and 99% sensitive to cefiderocol [60]. C-T, CAZ-AVI, IMI-REL, and cefiderocol all serve as potential empiric therapies for cUTI, cIAI’s, and nosocomial pneumonias caused by *P. aeruginosa*. The IDSA recommends the addition of metronidazole in cases of cIAI caused by *P. aeruginosa*. 

When sensitivities are known, a more targeted approach can be used. For MDR infections, piperacillin–tazobactam, ceftazidime, cefepime, aztreonam, or a fluoroquinolone (e.g., ciprofloxacin) is a suitable first-line choice if the *P. aeruginosa* isolate is sensitive. Carbapenems may be reserved as a second-line option for these infections. Traditional β-lactams that can be used to treat MDR infections are ineffective against DTR *Pseudomonas* because these strains are resistant. Newer agents such as C–T, CAZ–AVI, or IMI–REL would be the preferred first-line treatment options for DTR infections. MEV is not recommended as an antipseudomonal agent as its activity is no better than meropenem alone. Cefiderocol is considered a second-line agent for DTR *P. aeruginosa* due to current expert opinion favoring newer β-lactam/β-lactamase inhibitors. 

**Figure 3:**
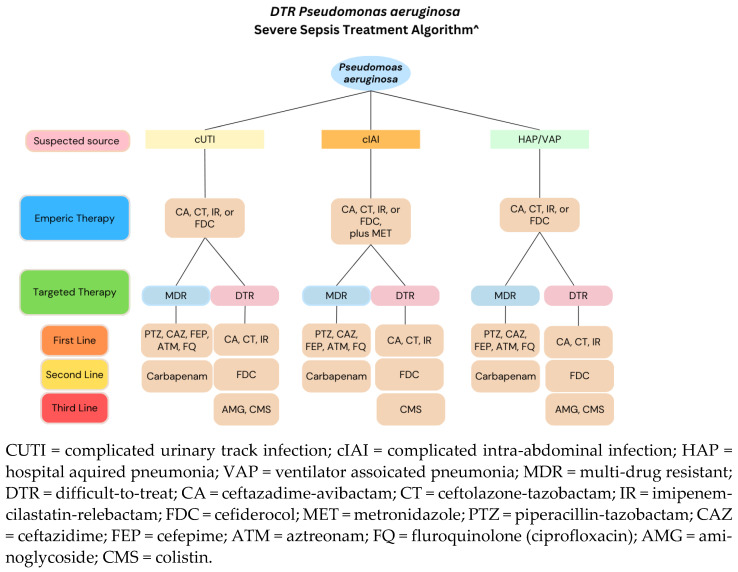
Possible treatment strategy for MDR/DTR *P. aeruginosa*. Adapted from [60].

If β-lactam agents alone are ineffective, tobramycin can be considered in combination with the use of a newer β-lactam (e.g., C–T, CAZ–AVI, IMI–REL, or cefiderocol). Colistin serves as a last-line agent due to its potential toxicities. Figure 3 depicts a possible treatment strategy for MDR/DTR *P. aeruginosa*. 

**Figure 4:**
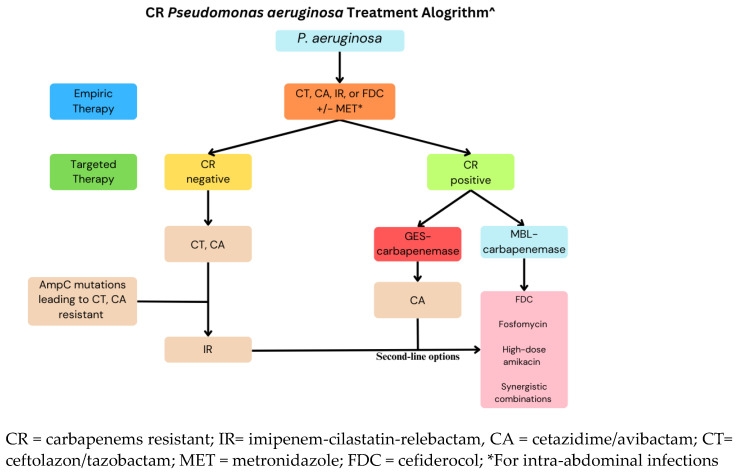
Proposed treatment of CR P. aeruginosa. Adapted from [60,94].

Figure 4 illustrates a proposed treatment of CR *P.aeruginosa* [60,94]. CAZ–AVI and C–T offer significant activity against non-CR strains. However, their effectiveness decreases in cases of resistance, such as in those strains with *AmpC* mutations. IMI-REL remains active despite *AmpC* overexpression and is a potential treatment in such cases. GES-producing strains show resistance to both C–T, and IMI–REL. Cefodericol is a potential treatment when all other therapies are ineffective, as it is stable against all carbapenamase (e.g., *GES*, *MBL*) and is unaffected by porin or efflux mutations. Fosfomycin can be effective, but its use as monotherapy is discouraged due to the risk of resistance. Synergistic combinations, such as colistin or aminoglycosides, are considered a last-resort treatment.

## 11. Future Directions 

Novel treatments are being developed as an alternative to traditional antibiotic methods. One of the strategies that has gained attention is bacteriophage therapy. Bacteriophages are viruses that infect bacteria and use their biology to replicate, eventually leading to the death of the bacterial cell. This alternative to antibiotics provides clinicians with the advantage of evolutionary tradeoffs. When bacteria’s genes are altered to resist phages, it can lead to changes in the genes responsible for antibiotic resistance mechanisms, potentially making the bacteria vulnerable to antibiotics [70].

New antibiotics are currently being developed to treat the MDR and DTR *P. aeruginosa*. Murepavadin is a drug that is currently in Phase 3 development and displays significant in vitro activity which includes carbapenemase-producing and colistin-resistant *P. aeruginosa.* The mechanism of action is an antimicrobial peptidomimetic with a novel, non-lytic mechanism of action [95].

## 12. Conclusions

MDR and DTR *P. aeruginosa* continue to be a rising public health threat and pose treatment challenges despite ongoing antimicrobial stewardship and drug development efforts. Recent advancements in rapid diagnostics now allow for the detection and diagnosis of *P. aeruginosa* in blood and the identification of gene-based resistance mechanisms. These developments enhance the ability to promptly initiate appropriate antipseudomonal therapy and help avoid adverse patient outcomes. A newer combination of Ceftolozane–tazobactam and imipenem–relebactam has improved activity against isolates of *Pseudomonas* resistant to the B-lactam alone. Cefiderocol is a potential treatment when all other therapies are ineffective, as it is stable against all carbapenemases (e.g., *GES, MBL*) and is unaffected by porin or efflux mutations. These newer antimicrobial agents have better safety profiles than classical antibiotics with comparable in vitro activity (Colistin, aminoglycosides). Clinicians should consider the limited data for *P. aeruginosa* infections when contemplating meropenem-vaborbactam as a treatment option. It is crucial to continue monitoring evolving antibiotic resistance patterns, as well as implementing surveillance programs for MDR *P. aeruginosa* strains and exploring novel approaches like phage therapy to combat this growing threat.

## Data Availability

No new data were created or analyzed in this review article. Data sharing is not applicable to this article.
